# Exploring the Therapeutic Potential of Green-Synthesized Gold Nanoparticles and *Ericaria selaginoides* Extract for Inflammatory Bowel Disease

**DOI:** 10.3390/antiox13080884

**Published:** 2024-07-23

**Authors:** Nayana Freire de Almeida Fontes, Mário Fernandes, Noelia González-Ballesteros, Maria Carmen Rodríguez-Argüelles, Andreia Castro Gomes, Antoniella Souza Gomes Duarte

**Affiliations:** 1 Departamento de Morfologia, Faculdade de Medicina, Centro de Ciências da Saúde, Universidade Federal do Ceará, Fortaleza 60440-900, Brazilantonielladuarte@ufc.br (A.S.G.D.); 2 Centre of Molecular and Environmental Biology (CBMA)/Aquatic Research Network (ARNET) Associate Laboratory, Universidade do Minho, Campus de Gualtar, 4710-057 Braga, Portugal; 3 Institute of Science and Innovation for Sustainability (IB-S), Universidade do Minho, Campus de Gualtar, 4710-057 Braga, Portugal; 4 Departamento de Química Inorgánica, Universidade de Vigo, 36310 Vigo, Spainmcarmen@uvigo.gal (M.C.R.-A.)

**Keywords:** inflammatory bowel disease, *Ericaria selaginoides*, macroalgae, gold nanoparticles, green synthesis, antioxidant, anti-inflammatory

## Abstract

Addressing disease remission and treatment adherence in inflammatory bowel diseases (IBDs), such as Crohn’s disease, poses significant challenges due to underlying oxidative and inflammatory processes. Nanotechnology emerges as a promising avenue for enhancing therapeutic outcomes in IBD by optimizing drug bioactivity, reducing toxicity, and extending circulation time. Gold nanoparticles, known for their resistance to gastrointestinal pH and possessing antioxidant and anti-inflammatory properties, offer particular promise. They can be produced by green synthesis with seaweed *Ericaria selaginoides* (ES), itself associated with gastroprotective and anti-inflammatory activities. In a murine model of Crohn’s disease induced with 8% acetic acid, pretreatment with dexamethasone (0.2 mL/30 g) or Au@ES (25 and 50 mg/kg) effectively mitigated inflammatory features. Notably, ES (50 mg/kg) and Au@ES (50 mg/kg) administration resulted in significant reductions in both macroscopic and microscopic inflammation scores compared to the disease control group. Furthermore, these treatments normalized inflammatory cytokine expression while safeguarding myenteric plexus glial cells. They also impeded neutrophil activation, leading to reduced myeloperoxidase activity and lipid peroxidation, coupled with increased glutathione levels. In conclusion, ES and Au@ES exhibit potent efficacy in counteracting inflammation and oxidation processes in an experimental Crohn’s disease model, suggesting their potential as alternative therapeutic strategies for IBD.

## 1. Introduction

More than 4.9 million people globally suffer from inflammatory bowel diseases (IBDs), which include Crohn’s disease and ulcerative colitis [1]. These are chronic idiopathic diseases that arise from a complex interplay of factors such as individual susceptibility, interactions with gut microflora, and the immune system’s role [2,3]. The dysfunction between effector and regulatory cells leads to an uncontrolled release of inflammatory molecules [4]. The enteric nervous system (ENS), composed of the myenteric plexus and the submucosal plexus, is a division of the nervous system, forming a neural network located in the wall of the organs of the gastrointestinal tract [5,6]. The neural network of the myenteric plexus is involved with the reflex regulation of the contractile activities of the external musculature, while the motor neurons of the submucosal plexus are related to the control of the secretomotor and vasomotor activities of the tunica mucosa [7].

Inflammation and oxidative stress are strongly interconnected. In IBD, oxidative stress is exacerbated due to the increased production of ROS/RNS by activated immune cells and impaired antioxidant defenses. Inflammation can induce oxidative stress through various mechanisms, including the activation of NADPH oxidases, nitric oxide synthases, and inflammatory pathways such as NF-kB. Conversely, oxidative stress can exacerbate inflammation by promoting the release of pro-inflammatory cytokines, activation of immune cells, and disruption of barrier function in the intestine [8]. Neutrophils and monocytes accumulate in the gastrointestinal wall and participate in IBD pathogenesis by producing inflammatory cytokines and soluble mediators such as ROS (e.g., neutrophil-myeloperoxidase (MPO) catalyzes the production of potent ROS) [9].

Current treatments involve pharmacotherapy, including immunosuppressants, with groundbreaking results seen with tumor necrosis factor (TNF) inhibitors [10]. However, challenges like non-response and loss of effectiveness have led to the exploration of emerging therapies like small molecules, apheresis, improved gut ecology, cell therapy, and exosomes. The ultimate aim is to achieve mucosal healing, linked to improved long-term outcomes [11]. Strategies that target the ENS are promising by potentially modulating gastrointestinal motility, maintaining mucosal barrier function, regulating immune responses, and restoring neuroimmune communication.

Nanotechnology has the potential to yield the most effective ways to deliver compounds or treat diseases directly, in a more controlled and safe manner, giving the possibility of the targeted delivery of molecules and accumulation of nanoparticles and bioactive compounds on the desired tissue [12]. Furthermore, it also facilitates overcoming certain barriers such as membranes and extreme pH [13], of particular relevance to IBD. Nanoparticles show promise in IBD therapy due to their small size, large surface area, and stability in the gastrointestinal tract. Various types, including polymeric, lipid-based, metallic nanoparticles, exosomes, and plant-derived nanoparticles, are being investigated for their potential in targeted drug delivery [14]. 

Gold nanoparticles (AuNPs) hold significant promise for treating IBD. Gold has a long history in therapy, thanks to its high biocompatibility and well-known antioxidant properties. With the advancements in nanotechnology, AuNPs have found applications not only in imaging but also in therapy [15]. They have been widely explored for treating various inflammatory conditions [16] such as neuroinflammation [17], autoimmune inflammation [18], and skin inflammation [19]. Numerous studies have highlighted their efficacy in managing inflammatory bowel diseases [19,20,21,22]. A notable advantage of AuNPs is their exceptional stability, especially at very low pH levels. This stability is crucial for their oral administration, enabling them to reach the intestine where they can exert their therapeutic effects on IBD pathology. This characteristic makes them particularly suitable for targeted interventions in IBD treatment [23].

Extracts from algae have already been used for alleviating IBD due to their potent anti-inflammatory properties. These are conferred by a high content in polysaccharides [24] which can counteract damage to the physical, chemical, immune, and biological barriers of the intestine [25]. Brown algae are especially promising for these applications since they have a polysaccharide content higher than 50% of their dry weight, with some species reaching values over 70% [6,26]. *Ericaria selaginoides* (ES), previously known as *Cystoseira tamariscifolia*, has a 50% content of polysaccharides, lower than some other closely related species [27], but its anti-inflammatory potential has been described to be higher than most algae species [28]. This high polysaccharide content also allows for the efficient production of gold nanoparticles by green synthesis, since the accumulation and reduction of gold ions is crucial for the obtention of these nanoparticles [29]. The green synthesis of gold nanoparticles in ES extract (Au@ES) was previously optimized and their cytocompatibility was validated [30]. The ES aqueous extract, rich in fucoidan and total phenolic content, revealed very high reducing and good scavenging activities [30].

In this study, we address the anti-inflammatory and antioxidant properties of ES and Au@ES in acetic acid-induced experimental colitis, a highly reproducible animal model. Macroscopic and histopathological data revealed that colitis induced by acetic acid causes the rupture of the superficial epithelium, multiple erosions, and mucosal ulcers, accompanied by a severe inflammatory reaction and abscess, thus resembling human colitis [31]. 

To our knowledge, this is the first study that addresses these properties in vivo in a murine model of IBD, which allows for a detailed evaluation of the positive effects observed upon oral administration of both the extract and derived nanoparticles. 

## 2. Materials and Methods

Preparation of the algae extract and gold nanoparticles: *Euricaria selaginoides* was collected at the lower intertidal rocky shore in the NW coast of Portugal (N 41 47.858′ W 008 52.423′). ES extract and Au@ES were prepared and characterized as previously reported [30]. As reported, Au@CT exhibited a mean diameter of 7.6 ± 2.2 nm and were spherical, polycrystalline, and stable.

Animals: The experiments used male Swiss mice (*Mus musculus*) 25 to 35 days old, with a body mass of 25 to 30 g, obtained from the Department of Physiology and Pharmacology-Universidade Federal do Ceará. The animals were kept in the vivarium of the Center for Studies in Microscopy and Image Processing, 6–8 animals per cage, at a temperature of 22–24 °C, in a 12 h light/dark circadian cycle, receiving standard feed and water and libitum. The standard food used was Nuvilab CR-1 rat and mouse food with the following nutritional composition: moisture (max) 125 g/kg, crude protein (min) 220 g/kg, ether extract (min) 50 g/kg, mineral material (max) 90 g/kg, raw fiber (max) 70 g/kg, calcium 10–14 g/kg, and phosphor 6000 mg/kg. The animals were fasted for 16 h before the experiments.

Experimental groups: A total of 75 animals were distributed in groups of 9 animals, for a total of 9 experimental groups, as in Table 1.

An experimental Crohn’s disease (CD) model was induced by the administration of acetic acid in mice.

The experimental protocol was based on previously published articles available in the literature, with modifications [31]. The animals were fasted for 12 h and given water ad libitum. To induce CD, 8% of acetic acid (AA) was diluted in distilled water (100 mL) and this solution was administered in a volume of 0.2 mL per 10 g of animal weight. The animals were previously anesthetized intraperitoneally (IP) with a mixture of ketamine (100 mg/kg) and xylazine (20 mg/kg) and placed in the left lateral decubitus position. A polyethylene catheter number 6 measuring 4 cm in length was inserted rectally to administer the 8% AA or saline solutions. Each animal remained suspended by the tail for 30 s to prevent the solutions from returning.

Microscopic and macroscopic evaluation: 12 h after inducing colitis, the animals were euthanized, followed by exsanguination. Using standard operating techniques, laparotomy was performed through a median incision, dissection and removal of the colonic segment for macroscopic evaluation. The specimens were opened longitudinally, washed with saline solution and distended on a flat surface. They were evaluated in a blinded manner according to the microscopic and macroscopic inflammation scores using a stereoscopic magnifying glass (Table 2) [32].

Determination of IL-1β and TNF-α cytokine levels: The levels of pro-inflammatory cytokines IL-1β and TNF-α present in the intestinal mucosa were quantified by ELISA. The best dose of EACP (3 mg/kg) was used to measure both. The collected tissues were homogenized in PBS 1X. The cytokines were detected using the DuoSet Kit (R&D Systems, Minneapolis, MN, USA), the primary antibodies for IL-1β and TNF-α were both acquired from Invitrogen. The 96-well ELISA plates were incubated with the capture antibody for 18 h at room temperature (100 μL of antibody per well). Subsequently, the plates were washed three times with 200 μL of wash buffer and blocked with 200 μL of 1% BSA for 1 h. After blocking, 100 μL of the samples or the standard curve preparations were added to each well and incubated for 2 h at room temperature. The plates were then washed three times with 200 μL of wash buffer and then incubated with cytokine detection antibody at room temperature for 2 h. After three washes, they were incubated with 100 μL of streptavidin at room temperature for 20 min. After another three washes, 100 μL of developer substrate solution was added to each well and incubated for 20 min at room temperature protected from light. The enzymatic reaction was stopped by adding 50 μL of stop solution (H_2_SO_4_). The absorbance was measured at 450 nm and the results were expressed in pg/mL.

Immunohistochemistry for S100 β and GFAP: To assess the localization of the inflammatory markers S100 β and GFAP in the intestinal mucosa, immunohistochemistry was carried out. Collected colon segments were isolated, separated and fixed in 10% buffered formalin for 20 h. The tissues were embedded in paraffin and cut into 4 μm thick cuts using a microtome, inserted into salinized histological slides. These slides were deparaffinized by oven at 60 °C overnight followed by three baths with xylene of five min each. The colons were then hydrated with two baths of absolute ethanol, one bath in 90% ethanol, one bath in 80% ethanol, and one bath in 70% ethanol, for three min each. The sections were submerged in a distilled water bath for 10 min and antigen recovery was carried out with citrate buffer (DAKO, Singapore; pH 7.0) for 20 min in a water bath (95 °C). The tissues were washed with phosphate-buffered saline (PBS 1X) for 5 min after which the peroxidase was blocked with 3% hydrogen peroxide (Abcam, Oxford, UK) for 30 min. The slides were washed with PBS 1X and incubated with the primary antibodies for S100 beta and GFAP (both from DAKO) for 1 h at room temperature. In the negative controls, the primary antibodies were omitted. The slides were washed three times with PBS 1X and incubated with the polymer (DAKO) for 30 min. The slides were then washed three times with PBS 1X for three minutes each, dried and DAB was applied (DAKO, 3,3-diaminobenzidine, one drop of DAB to 1 mL of diluent). DAB is a chromogen that reacts with the peroxidase of the target antigen, resulting in a brown color. In this way, the slides were observed until a brown coloration appeared, after which the reaction was stopped immediately by immersing them in distilled H_2_O. Finally, the slides were counterstained with Mayer’s hematoxylin and processed to insert the coverslip. The immunohistochemical images were captured (eight fields per slide for each animal) using a light microscope coupled to a camera with a LAZ 3.5 acquisition system (LEICA DM1000, Wetzlar, Germany). To quantify the number of cells positive for S100 beta and GFAP, the Image J program (version 1.53k) was used. The results were expressed as the mean ± SEM of the number of cells positive for GFAP or S100 beta per field.

Evaluation of Myeloperoxidase Activity (MPO): For this assay, 50 to 100 mg of colon from each animal was placed in buffer 0.1 M NaCl + 0.015 M Disodium EDTA and 0.02 M Na_3_PO_4_ at pH 4.7. After homogenization in a Polytron at 18,516 g, they were centrifuged for an additional 15 min at 986 g. The supernatant was then removed, the precipitate was resuspended in 0.1 M NaCl + 0.015 M Disodium EDTA and 0.02 M Na_3_PO_4_ at pH 4.7, and it was again centrifuged for 15 min at 986 g. The supernatant was removed, and the precipitate was homogenized in a Politron at 18,516 g in HTAB 0.05% diluted in 200 mL of Na_3_PO_4_ 0.05 M. This homogenate was then frozen and thawed in liquid nitrogen twice. The homogenate was centrifuged at 10,956–21,475 g for 15 min and the supernatant was pipetted onto a plate (5–10 μL) to which 45 μL of 0.08 M Na_3_PO_4_ was added. Then, we added 25 μL of TMB and 100 μL of H_2_O_2_ for 5 min. The reaction was completed with the addition of 50 μL of a 4 M of H_2_SO_4_ and the absorbance were read on a plate reader at 450 nm (FLUOstar OPTIMA-BMG LABTECH). The neutrophil infiltrate was obtained from a neutrophil standard curve and the results are expressed as MPO units per mg tissue.

Determination of Malondialdehyde (MDA) Levels: MDA levels were determined using the method of Uchiyama and Mihara [33]. An amount of 50 mg of intestinal mucosa tissue samples corresponding to the colon was homogenized in 500 µL of 1.15% KCl solution at 4 °C, to obtain a 10% homogenate. From this, 250 µL aliquots of the homogenate were added to tubes containing 1.5 mL of 1% H_3_PO_4_ solution and 500 µL of TBARS solution (0.6%). The tubes were heated in a water bath at 100 °C for 45 min, then cooled in an ice-cold water bath, followed by the addition of 2 mL of n-butanol. The samples were then vortexed (PHOENIX Instrument, Garbsen, Germany) for 1 min and then centrifuged at 158 g for 15 min. The supernatant absorbance was measured at 520 and 535 nm in a plate reader (FLUOstar OPTIMA - BMG LABTECH, Offenburg, Germany), and the results correspond to the difference between absorbances which were converted to nmol/g of intestinal tissue.

Determination of Glutathione (GSH) levels: The colon samples were homogenized in 0.02 M EDTA (1 mL/100 mg tissue). Subsequently, 400 μL of homogenate was mixed with 300 μL of distilled water and 80 μL of trichloroacetic acid (50%, *w*/*v*) and centrifuged at 986 g for 15 min. Then, 400 μL of supernatant was mixed with 800 μL of Tris buffer (0.4 M, pH 8.9), 20 μL of 0.01 M DTNB was added, and the samples were subsequently stirred for a period of 3 min. A reading spectrophotometer with absorbance adjustment measured at 412 nm was used. The results are expressed as μg of GSH/g tissue.

Statistical analysis: All quantitative results were expressed as the mean ± standard deviation (SD), except for the histopathology scores, which were expressed as the median. The data were statistically analyzed using GraphPad Prism^®^ software, version 5.0. The following tests were used to carry out the statistical analysis between the groups: analysis of variance (ANOVA) followed by Bonferroni’s multiple comparison test, and for the histological scores, the Kruskal–Wallis test followed by Dunn’s test. The significance level adopted was 5% (*p* < 0.05) and descriptive levels (*p*) below this value were considered significant.

## 3. Results

### 3.1. Ericaria selaginoides Extract and Gold Nanoparticles Improve the Macroscopic Appearance of Murine Colon in Induced Colitis Model

**Figure 1:**
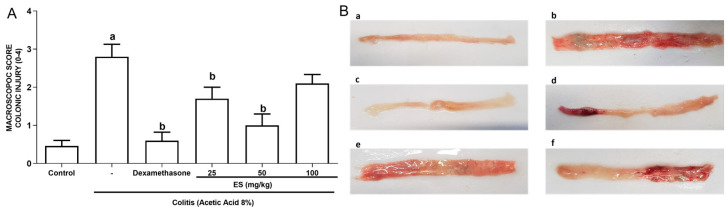
(**A**) Macroscopic evaluation of inflammatory activity (total score) in the colon of mice treated with the extract of the algae *Ericaria selaginoides* (ES), at the dose of 25 (n = 6), 50 (n = 6), and 100 mg/kg (n = 6) or dexamethasone (n = 6) in the experimental colitis model using 8% acetic acid (8% AA; n = 6), according to the criteria of [32]. The columns represent the mean ± SEM of the total macroscopic scores, analyzed by the one-way ANOVA test followed by the Bonferroni test. ^a^ *p* < 0.05 vs. control group; ^b^ *p* < 0.05 vs. 8% AA group. (**B**) Images depicting the macroscopic appearance of the colon of mice treated with the extract of ES in the model of experimental colitis caused by 8% AA: (**a**) control group, (**b**) colitis group, (**c**) dexamethasone group, (**d**) ES 25 mg/kg group, (**e**) ES 50 mg/kg group, and (**f**) ES 100 mg/kg group.

To address the alleviation of the macroscopic effects of AA-induced colitis by *E. selaginoides* (ES) extract and gold nanoparticles synthesized in it (Au@ES), the colon was observed after the animals were euthanized. The independent observation under a stereoscopic magnifier revealed that there was a significant increase in the macroscopic scores of inflammations in the colitis group, 12 h after the induction of colitis by 8% AA, when compared to the control and dexamethasone groups (Figure 1 and Table 3). This elevated inflammation translates into severe colonic mucosal injury and macroscopic edematous colonic inflammation. Analysis of the results identifies that 25 and 50 mg/kg of ES extract were able to significantly reduce the total inflammatory parameters when compared to the colitis group. There was no difference between the scores obtained with ES 100 mg/kg when these mice were compared to the colitis group, indicating that this higher concentration may induce some level of toxicity, impairing the recovery of the colon affected by AA-induced colitis.

**Figure 2:**
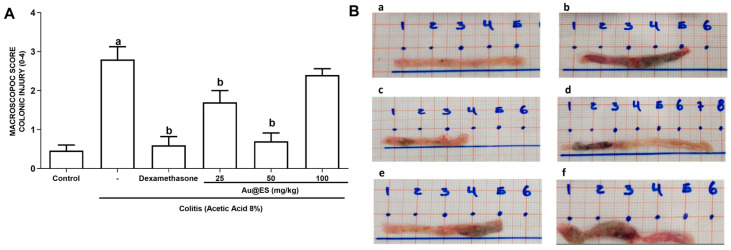
(**A**) Macroscopic evaluation of inflammatory activity (total score) in the colon of mice with experimental colitis treated with Au@ES, at the dose of 25 (n = 6), 50 (n = 6), and 100 mg/kg (n = 6) or dexamethasone (n = 6) in the experimental colitis model using 8% acetic acid (8% AA; n = 6), according to the criteria of [7]. The columns represent the mean ± SEM of the total macroscopic scores, analyzed by the one-way ANOVA test followed by the Bonferroni test. ^a^ *p* < 0.05 vs. control group; ^b^ *p* < 0.05 vs. 8% AA group. (**B**) Images depicting the macroscopic appearance of the colon of mice with induced colitis treated with Au@ES: (**a**) control group, (**b**) colitis group, (**c**) dexamethasone group, (**d**) Au@ES 25 mg/kg group, (**e**) Au@ES 50 mg/kg group, and (**f**) Au@ES 100 mg/kg group.

A similar assay was performed for Au@ES. According to the macroscopic scores observed, pretreatment with Au@ES protected the colon from lesions induced by AA, at doses of 25 and 50 mg/kg, when compared to the colitis group. This result was similar to that observed with the standard drug dexamethasone. There was no significant difference in results obtained with Au@ES 100 mg/kg and the colitis group (Figure 2 and Table 4).

Pretreatment with dexamethasone (Figure 2B(c)) or Au@ES, at doses of 25 and 50 mg/kg, prevented inflammatory aspects such as ulcers, hyperemia, edema and prolapse of the colon, when compared to the Colitis group (Figure 2B(b)). Au@ES 100 mg/kg (Figure 2B(f)) did not induce an effect similar to that obtained with 25 and 50 mg/kg doses.

### 3.2. Ericaria selaginoides Extract and Gold Nanoparticles Improve the Microscopic Hallmarks of Inflammation

**Figure 3:**
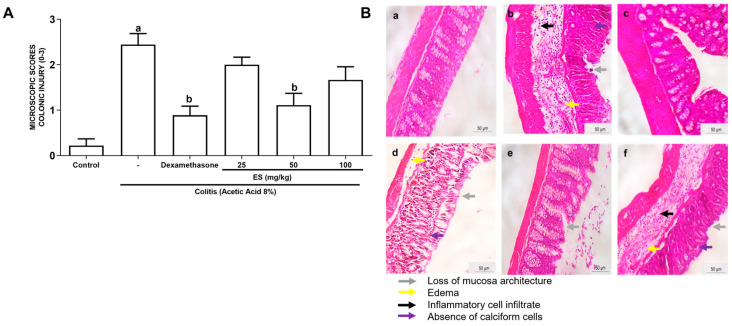
(**A**) Microscopic assessment of inflammatory activity (total score), according to Appleyard’s criteria [8]. Control group (n = 6), colitis control group (n = 6), dexamethasone group (n = 6), mice treated with ES extract: 25 mg/kg (n = 6), 50 mg/kg (n = 6), and 100 mg/kg (n = 6). The columns represent the mean ± SEM of the microscopic scores, analyzed by the one-way ANOVA test followed by the Bonferroni test. ^a^ *p* < 0.05 vs. control group; ^b^ *p* < 0.05 vs. 8% AA group. (**B**) Hematoxilin–eosin-stained colon of mice with induced colitis treated with ES. Size bar corresponds to 50 µm. Control group (**a**), colitis control group (**b**), dexamethasone-treated mice (**c**), mice treated with ES extract: 25 mg/kg (**d**), 50 mg/kg (**e**), and 100 mg/kg (**f**).

In the microscopic analysis of total inflammation scores according to the criteria of [8], it was observed that 8% AA promoted important alterations such as hemorrhagic lesion, edema, loss of architecture, and infiltration of inflammatory cells in the colon of the animals studied when compared to the control group. Oral pretreatment with ES at a dose of 50 mg/kg significantly decreased the microscopic scores mentioned above. Similar results were observed in the dexamethasone group (Figure 3 and Table 5).

**Figure 4:**
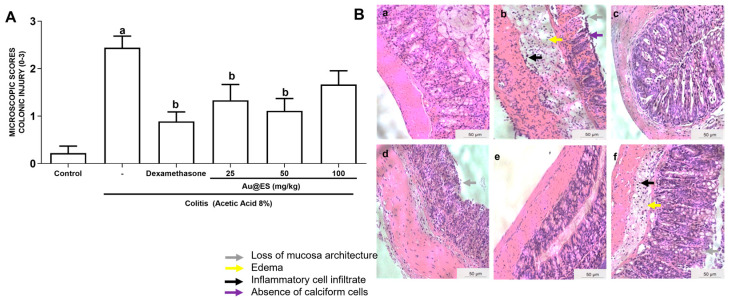
(**A**) Microscopic assessment of inflammatory activity (total score), according to [8]. Control group (n = 6), colitis control group (n = 6), dexamethasone group (n = 6), and mice treated with Au@ES extract: 25 mg/kg (n = 6), 50 mg/kg (n = 6), and 100 mg/kg (n = 6). The columns represent the mean ± SEM of the microscopic scores, analyzed by the one-way ANOVA test followed by the Bonferroni test. ^a^ *p* < 0.05 vs. control group; ^b^ *p* < 0.05 vs. 8% AA group. (**B**) Hematoxilin–eosin-stained colon of mice suffering from experimental colitis treated with Au@ES. Size bar corresponds to µm. Control group (**a**), colitis control group (**b**), dexamethasone-treated mice (**c**), mice treated with Au@ES: 25 mg/kg (**d**), 50 mg/kg (**e**), and 100 mg/kg (**f**).

Figure 4 illustrates the results of the microscopic evaluation, showing the intense inflammatory process afflicting the animals in the colitis group, with the presence of cellular infiltrate, loss of mucosal architecture, hemorrhage, and the depletion of goblet cells (Figure 4B(b)) when compared to the control group (Figure 4B(a)). In the control group Figure 4B(a)) and dexamethasone group (Figure 4B(c)), a normal colonic appearance was observed. An improvement in the inflammatory aspects induced by AA was observed in animals which received 50 mg/kg of ES orally (Figure 4B(e)). The ES 25 and 100 mg/kg groups did not prevent the damaging effect of AA, showing an inflammatory pattern similar to the animals with colitis (Figure 4B(d) and Figure 4B(f), respectively).

As seen in Figure 4A and Table 6, AA promoted important microscopic alterations such as hemorrhagic lesions, edema, loss of architecture, and infiltration of inflammatory cells in the colon of the animals studied when compared to the control group. Oral pretreatment with Au@ES at doses of 25 and 50 mg/kg reduced the microscopic scores (*p* < 0.05). These data were similar to those observed in the dexamethasone group. No statistical difference was observed at a dose of 100 mg/kg when compared to the colitis group.

Figure 4B illustrates the results of the microscopic evaluation, showing the intense inflammatory process in the animals in the colitis group, with the presence of cellular infiltrates, loss of mucosal architecture, hemorrhage, and depletion of goblet cells (Figure 4B(b)) when compared to the control group (Figure 4B(a)). In the control group (Figure 4B(a)) and the dexamethasone group (Figure 4B(c)), a normal colonic appearance was apparent. An improvement in the inflammatory markers induced by AA was observed in animals pretreated with 25 and 50 mg/kg Au@ES orally (Figure 4B(d) and Figure 4B(e), respectively). The Au@ES 100 mg/kg group did not benefit from any such improvement, showing an inflammatory pattern in the colon similar to that of animals with induced colitis.

### 3.3. Neutrophil Activation Is Affected by Administration of Ericaria selaginoides Extract and Gold Nanoparticles

**Figure 5:**
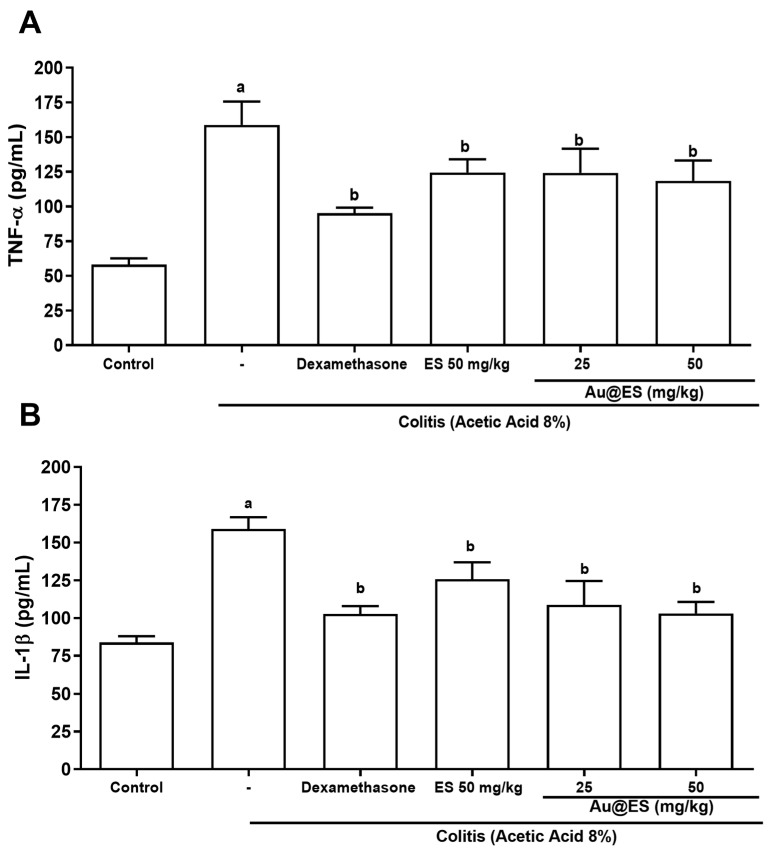
Treatments with ES at the dose of 50 mg/kg (n = 6) and Au@ES at the dose of 25 (n = 6) and 50 mg/kg (n = 6) or dexamethasone (n = 6) decreased tissue levels of inflammatory mediators TNF-α (**A**) and IL-1β (**B**) in experimental colitis (n = 6). The columns represent the mean ± SEM of cytokine levels, expressed in pg/mL. Data were analyzed using the one-way ANOVA test followed by the Bonferroni test. ^a^ *p* < 0.05 vs. control group; ^b^ *p* < 0.05 vs. 8% AA group.

The colonic samples were submitted to an assay to determine the levels of the pro-inflammatory cytokine TNF-α (pg/mL). The animals in the 8% AA group showed a significant increase in TNF-α levels (158.71 ± 16.9) compared to the control group (58.14 ± 4.4). ES 50 mg/kg (124.4 ± 9.6) and Au@ES at doses of 25 (121.6 ± 17.4) and 50 mg/kg (118.5 ± 14.54), as well as dexamethasone (95.24 ± 3.8), decreased the level of this cytokine when compared to the colitis group (Figure 5).

**Figure 6:**
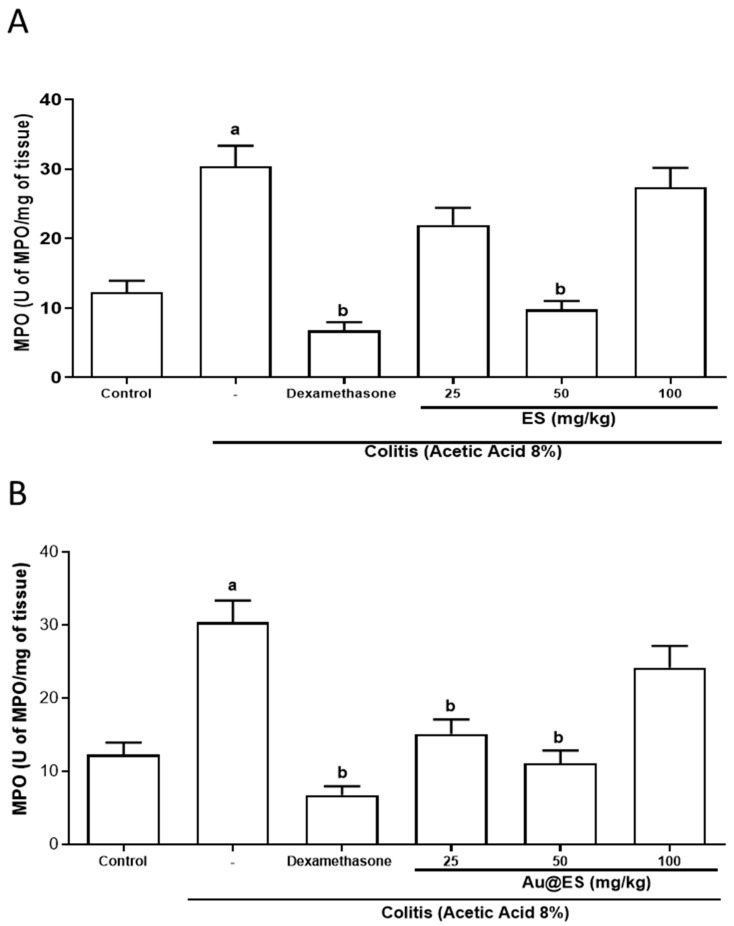
Treatments with ES (**A**) at the dose of 50 mg/kg (n = 6) and Au@ES (**B**) at the dose of 25 (n = 6) and 50 mg/kg (n = 6) or dexamethasone (n = 6) decreased on myeloperoxidase (MPO) levels in the colon of mice in experimental colitis (n = 6). The columns represent the mean ± SEM of cytokine levels, expressed MPO/mg of tissue. Data were analyzed using the one-way ANOVA test followed by the Bonferroni test. ^a^ *p* < 0.05 vs. control group; ^b^ *p* < 0.05 vs. 8% AA group.

The activity of the enzyme myeloperoxidase (MPO) was evaluated as a parameter to verify the anti-inflammatory activity of ES extract by regulating neutrophil infiltration. Figure 6A and Supplementary Table S1 show that the animals in the colitis group showed increased MPO activity (MPO unit/mg tissue) compared to the group of animals treated with saline (30.43 ± 2.9 vs. 10.65 ± 1.3). ES 50 mg/kg (9.77 ± 1.2) and dexamethasone (11.76 ± 2.1) significantly reduced neutrophil-produced myeloperoxidase when compared to the colitis group (30.43 ± 2.9). However, the doses of 25 (21.97 ± 2.4) and 100 (27.36 ± 2.8) mg/kg ES did not reverse the altered MPO levels in colitis. Interestingly, Au@ES at doses of 25 (15.13 ± 1.9) and 50 mg/kg (11.11 ± 1.7) significantly decreased neutrophil infiltration when compared to the colitis group (30.43 ± 2.9). However, the 100 (29.19 ± 2.9) mg/kg dose of Au@ES did not reverse the MPO increase induced by AA (Figure 6B and Supplementary Table S1).

### 3.4. ES Extract and Au@ES Counteract Oxidative Events Associated with Induced Colitis

**Figure 7:**
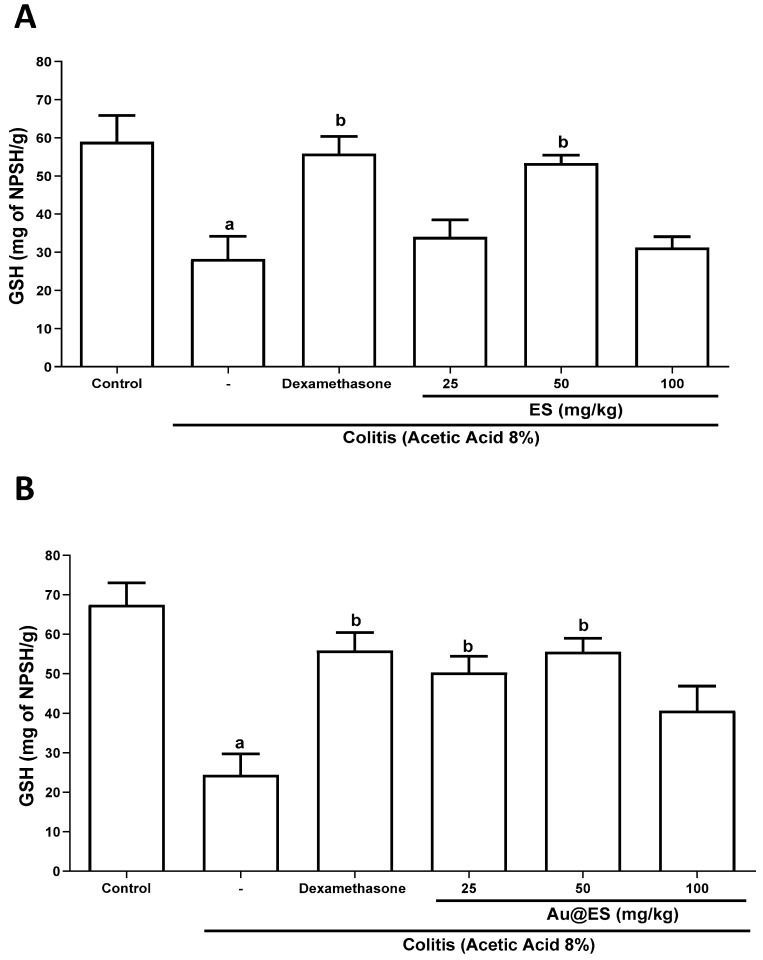
Effect of ES (upper panel) (**A**), at the dose of 50 mg/kg (n = 6), and Au@ES (lower panel) (**B**), at the dose of 25 (n = 6) and 50 mg/kg (n = 6) or dexamethasone (n = 6) on reduced glutathione (GSH) levels in the colon of mice subjected to the experimental colitis model using 8% AA (n = 6). Columns represent the mean ± SEM of GSH levels, expressed as mg NPSH/g tissue. Data were analyzed using the one-way ANOVA test followed by the Bonferroni test. ^a^ *p* < 0.05 vs. control group; ^b^ *p* < 0.05 vs. AA group.

The concentration of reduced glutathione levels (mg of NPSH/g of tissue) in the colon of mice after AA administration decreased significantly when compared to control group mice (28.24 ± 5.9 vs. 58.92 ± 6.9). Pretreatment with either ES, at a dose of 50 mg/kg (53.34 ± 2.1), or dexamethasone (55.86 ± 4.5) proved effective in restoring the values of reduced glutathione. The doses of 25 (34.04 ± 4.4) and 100 (31.22 ± 2.8) mg/kg of ES did not influence this parameter (Figure 7A and Supplementary Table S2). Interestingly, pretreatment with Au@ES, at doses of 25 (50.29 ± 4.1) and 50 (55.55 ± 3.8) mg/kg, restored normal GSH levels when compared to the colitis group, while Au@ES at a dose of 100 (40.67 ± 6.1) mg/kg did not (Figure 7B and Supplementary Table S2).

**Figure 8:**
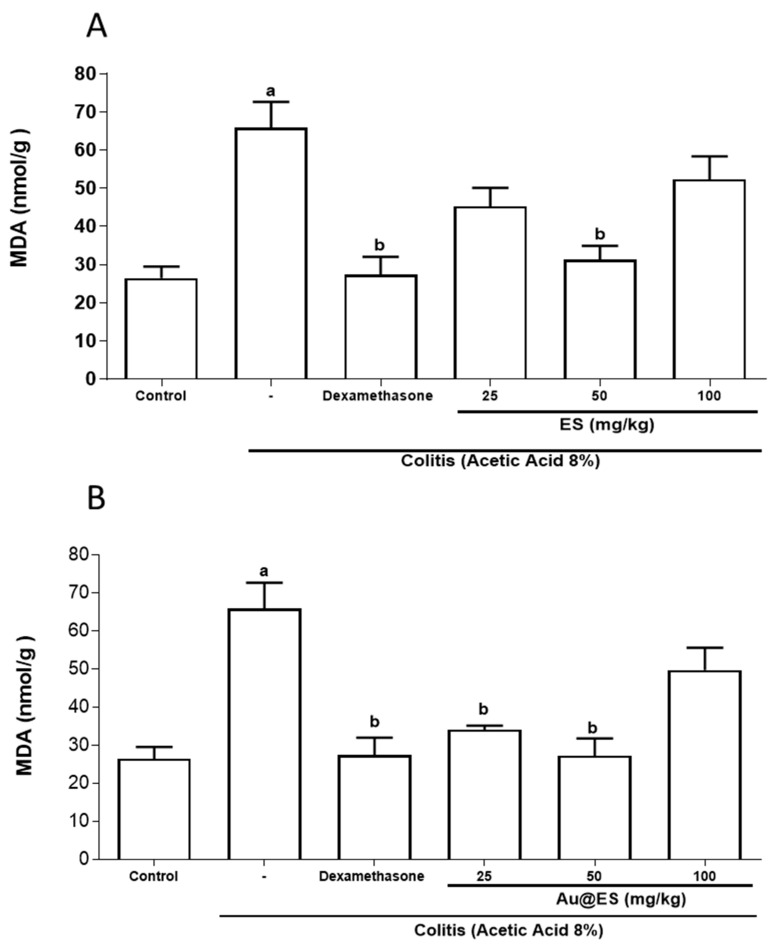
Effect of ES (upper panel) (**A**), at the dose of 50 mg/kg (n = 6), and Au@ES (lower panel) (**B**), at the dose of 25 (n = 6) and 50 mg/kg (n = 6) or dexamethasone (n = 6) on malondialdehyde (MDA) levels in the colon of mice with AA-induced colitis (n = 6). Columns represent the mean ± SEM of MDA levels, expressed as nmol/g tissue. Data were analyzed using the one-way ANOVA test followed by the Bonferroni test. ^a^ *p* < 0.05 vs. control group; ^b^ *p* < 0.05 vs. 8% AA group.

In Figure 8A and Supplementary Table S3, it is evidenced that colitis caused by AA increased MDA levels (1715 ± 239.4 nmol/g tissue) when compared to the control group (910 ± 101.4), indicating a severe process of lipid peroxidation and thereby underlying striking oxidative stress. ES proved to have an important antioxidant effect in this experimental colitis model, especially at a dose of 50 mg/kg (1024 ± 115.1), with which we observed a significant reduction in MDA levels when compared to the colitis group. A similar result was observed in the dexamethasone group (1110 ± 49.19), when compared to the colitis group. However, the doses of 25 (1380 ± 123.8) and 100 (1320 ± 125.4) mg/kg of *E. selaginoides* did not reverse the increase in lipid peroxidation observed in mice with colitis. In Figure 8B and Supplementary Table S3, it is shown that Au@ES at doses of 25 and 50 mg/kg were able to significantly prevent lipid peroxidation induced by AA. The colon of mice given Au@ES at a dose of 100 mg/kg showed high levels of MDA when compared to those treated with smaller dosages of Au@ES.

### 3.5. Myenteric Plexus Markers Altered in Induced Colitis Are Normalized with ES and Au@ES

**Figure 9:**
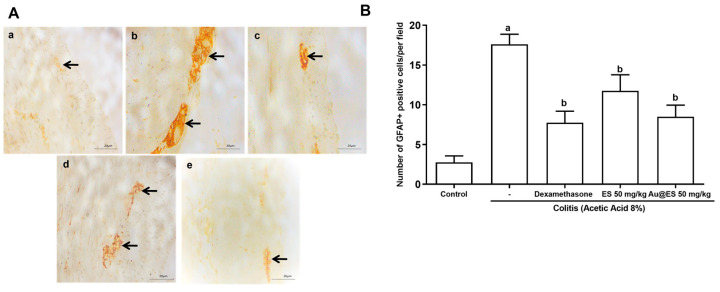
(**A**) Detection of GFAP-positive cells (as marked by arrows) in myenteric plexus in colon of mice with AA-induced colitis. Control group (**a**), colitis control (**b**), dexamethasone-treated mice (**c**), mice treated with ES 50 mg/kg (**d**), or Au@ES 50 mg/kg (**e**). (**B**) ES 50 mg/kg (n = 6) and Au@ES 50 mg/kg (n = 6) decrease the number of cells immunostained for GFAP in experimental colitis (n = 6). The columns represent the mean ± SEM of immunostained cells, expressed as the number of GFAP+ positive cells per field. Data were analyzed using the one-way ANOVA test followed by the Bonferroni test. ^a^ *p* < 0.05 vs. control group; ^b^ *p* < 0.05 vs. 8% AA.

In the experimental model of colitis induced with 8% AA, a significant number of glial cells present in the myenteric plexus expressed GFAP (Figure 9A(b)) when compared to the control group (Figure 9A(a)). Based on the photomicrographs presented, pretreatment with ES 50 mg/kg (Figure 9A(d)) and Au@ES 50 mg/kg (Figure 9A(e)) decreased GFAP immunolabeling in the myenteric plexus of animals suffering from colitis. This result was similar to that obtained with dexamethasone (Figure 9A(c)). Quantitatively, immunolabeling for GFAP (Figure 9B) was increased in the colitis group (17.63 ± 3.76) when compared to the control group (2.75 ± 0.81). ES 50 mg/kg (11.75 ± 2.02) and Au@ES 50 mg/kg (8.53 ± 1.45), as well as dexamethasone (7.71 ± 1.41), decreased the labeling (*p* < 0.05) when compared to the colitis group, in terms of GFAP-positive cells per field.

**Figure 10:**
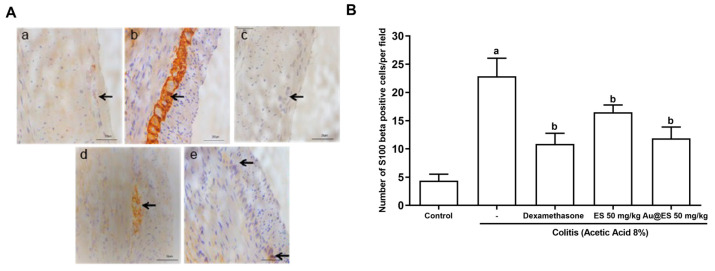
(**A**) Detection of S100 β-positive cells (as marked with arrows) in myenteric plexus of mice with experimental colitis. Control group (**a**), colitis control (**b**), dexamethasone-treated mice (**c**), mice treated with ES 50 mg/kg (**d**), or Au@ES 50 mg/kg (**e**). (**B**) Both ES 50 mg/kg (n = 6) and Au@ES 50 mg/kg (n = 6) decrease the number of cells immunostained for S100-β in experimental colitis (n = 6). The columns represent the mean of GFAP+ positive cells per field SEM. Data were analyzed using the one-way ANOVA test followed by the Bonferroni test. ^a^ *p* < 0.05 vs. control group; ^b^ *p* < 0.05 vs. 8% AA group.

In Figure 10A, it is possible to observe an increase in the number of glial cells present in the myenteric plexus immunolabeled for S100 β in the colitis group (Figure 10A(b)), when compared to the control group (Figure 10A(a)). Pretreatment with dexamethasone (Figure 10A(c)), ES 50 mg/kg (Figure 10A(d)), and Au@ES 50 mg/kg (Figure 10A(e)) decreased the immunolabeling of S100 beta in the myenteric plexus of animals submitted to AA-induced colitis. Figure 10B shows a significant quantitative increase in S100 beta labeling in the colitis group (22.88 ± 3.18) compared to the control group (4.87 ± 1.74). Dexamethasone (10.8 ± 2.9), ES 50 mg/kg (16.50 ± 2.87), and Au@ES 50 mg/kg (11.88 ± 2.04) decreased the number of S100 β-positive cells per field (*p* < 0.05) when compared to the colitis group.

## 4. Discussion

Inducing colitis in rodents using a chemical agent, such as AA, offers the advantage of instigating inflammation in animals with a normal immune system [34]. Rectally administered AA can replicate colitis in a diffuse and dose-dependent manner, observed in the distal portion of the rodent colon [35,36]. This induces inflammation akin to ulcerative colitis and Crohn’s disease in humans, considering molecular changes, histological features, and clinical characteristics [37]. Our findings align with the existing literature, revealing an increase in macroscopic lesions in the colons of rats, 12 h after the administration of 8% AA [38], which could be treated with dexamethasone as a control treatment.

The administration of ES demonstrated a reduction in overall inflammatory aspects of AA-induced colitis, in terms of macro- and microscopic scoring, most significantly at 50 mg/kg. Interestingly, with 25 and 50 mg/kg Au@ES, the animals also significantly ameliorated in terms of inflammation scores and histology markers. However, Au@ET 100 mg/kg did not exhibit a similar effect. It is noteworthy that ES and Au@ES yielded comparable results to the standard drug, dexamethasone.

ES is a brown macroalgae rich in polysaccharides and phenolic compounds [39]. Therefore, it has been associated with anti-inflammatory [28] and antioxidant activities [27]. As previously reported by our group, ES aqueous extract has a high content in phenolic compounds, revealing extremely high reducing activity and good free radical scavenging capacity [30]. This observation was in line with characterization data of extracts with organic solvents [27,40]. Together, they support the concept that ES can directly counteract oxidative stress and mitigate oxidative-mediated damage to biomacromolecules. This antioxidant activity facilitates the green synthesis of metallic nanoparticles in aqueous extracts of this algae [30,41]. Macroalgae polysaccharides are considered a potential therapeutic option for patients with IBD [4]. Sulfated polysaccharides from the macroalgae *Gracilaria caudata* have been shown to improve macro- and microscopic scores of histopathology of colitis, induced with ethanol, in mice [42]. A similar positive effect on colon damage was observed with polysaccharide extracted from red algae *Gracilaria birdiae* and *Hypnea musciformin* on trinitrobenzenesulfonic acid (TNBS)-induced colitis [43,44]. Interestingly, the application of naked AuNPs on rodent models of colitis has also reportedly led to the improved status of the colon [45].

A key feature of AA-induced colitis is immune cell infiltration, producing high levels of proinflammatory cytokines such as TNF-α and IL-1β [46]. These chemoattractant mediators are essential for cytokine-dependent neutrophil activation, associated with elevated MPO levels. In this study, animals with colitis induced by AA displayed a notable increase in the concentration of both IL-1β and TNF-α, assessed through ELISA and immunohistochemistry, as reported by others [38]. Administration of dexamethasone, ES at a dose of 50 mg/kg, and Au@ES at doses of 25 mg/kg and 50 mg/kg leads to a significant reduction in the expression of these proinflammatory cytokines in the colon of animals with induced IBD. This observation suggests that the protective effect of ES and Au@ES may be mediated by hampering the inflammatory process. This is in agreement with reports linking the administration of algal extracts or components (mostly polysaccharides, e.g., fucoidan), particularly brown algae, to the diminished expression of these inflammatory mediators [24,46,47]. A previous study proved that the ethanolic extract of ES had some anti-inflammatory properties, with a better activity in extracts collected in the summer [28]. Sulfated polysaccharides of related species of the *Cystoseira* genus, formerly the genus of ES, are recognized for their anti-inflammatory, gastroprotective, and antiradical activities [47]. Mhadhbei et al. showed that aqueous extracts of *Cystoseira amentacea, Cystoseira crinita, Cystoseira sedoides,* and *Cystoseira compressa* reduced inflammation in rat paw oedema, with doses of 25 mg/kg and 50 mg/kg, a few hours after incubation [48]. The concentrations used, although in the context of other clinical applications, are comparable to the ones used by our group, indicating that this family of brown algae has potential for multiple applications in inflammatory diseases. With *Sargassum hemiphyllum*, the capacity to inhibit the expression of IL-1β and TNF-α, while promoting IL-10 and IFN-γ release, was directly associated with better intestinal epithelial barrier and immune function [49]. Polysaccharides from *G. caudata* also improved the disease status in AA-induced colitis by regulating the production of these inflammation mediators [38]. Interestingly, in clinical practice, the inhibition of TNF-α serves as a widely adopted standard treatment for IBD [50], emphasizing the importance of finding natural products with a similar activity.

AuNPs have also been associated with reduced inflammation in chronic diseases, with a particular emphasis on colitis. AuNPs have been shown to attenuate colonic inflammation, reduce oxidative stress, inhibit pro-inflammatory cytokines, and promote tissue repair in experimental models of colitis [9]. Other studies also proved that the expression of interleukin-17, a key mediator in the pathogenesis of intestinal inflammation, was sensitive to AuNPs [20]. Notably, AuNPs produced by green synthesis in algae have already been used to suppress cytokine production in vitro [51]. AuNPs produced in extracts of three seaweeds, *C. myrica*, *C. trinodis*, and *C. prolifera*, were also able to diminish inflammation in vitro by inhibiting egg albumin denaturation [52]. Curiously, Zhu et al. proved that the incubation of monocytes with AuNPs for 5 h resulted in a significant reduction in ROS-mediated activation of the inflammation signaling pathway [22]. As the current study did not aim to draw a comprehensive profile of the modified cytokine expression patterns, it would be important in future studies to clarify if ES and Au@ES can interfere with IL17-driven inflammatory processes, and even upstream cytokines IL12 and IL23, emergent therapeutic targets in gut inflammation [53].

In IBD, oxidative stress is exacerbated due to the increased production of ROS/RNS by activated immune cells and impaired antioxidant defenses. This can cause oxidative damage to cellular components such as lipids, proteins, and DNA. Conversely, oxidative stress can exacerbate inflammation by promoting the release of pro-inflammatory cytokines, activation of immune cells, and disruption of barrier function in the intestine. Neutrophil activation and infiltration in AA-induced disease [54] reflect the importance of this feature in IBD. These cells release granular MPO in the inflamed bowel, which leads to the production of powerful ROS and perpetuates inflammation [55]. MPO is thus considered a biomarker for IBD. Colons from mice treated with ES 50 mg/kg and Au@ES 25 or 50 mg/kg displayed a striking impairment of neutrophil activation, with significantly lowered MPO activity, and consequently very reduced lipid peroxidation and augmented glutathione levels. This observation is in agreement with the already discussed antiradical activity of ES [27,30,40] but also hints at the concomitant induction of antioxidant enzymatic defenses. This strong antioxidant activity, unquestionably beneficial to interrupt the pathological cascade in IBD, is comparable to that of purified sulfated polysaccharides from *G. caudata* in AA-induced and from *G. birdiae* in TNBS-induced models, previously cited [38]. The first was effective at 1.0 to 10.0 mg/kg administered intraperitoneally, which is very invasive, while the latter showed positive results at 30, 60, and 90 mg/kg by oral administration, more approximate to the current study.

The lower dose of Au@ES (25 mg/kg) leading to a maximal effect on antioxidant biomarkers is possibly related to the antiradical activity of AuNPs. Orally administered gold nanoclusters showed ROS scavenging, with a consequent reduction in the expression of proinflammatory cytokines, beneficial for inflammatory bowel disease treatment [23]. Oral administration of naked AuNPs (2.5 mg Au/kg) was also shown to effectively target colonic tissue in dextran sodium sulphate (DSS)-induced ulcerative colitis, reducing lipid peroxidation and displaying anti-inflammatory potential [20]. Interestingly, the current work used similar dosages of gold (25 mg/kg of Au@ES containing 3.4 mg Au/kg). Another study reported that orally given AuNPs (25 μg Au/kg) can prevent colitis by attenuating the inflammatory response mediated by Toll-like receptor 4 and oxidative stress but may lead to an imbalance of the intestinal flora of mice [22]. The green synthesis of AuNPs as in the present study may mitigate the risk of dysbiosis, as macroalgae are considered a source of prebiotics [56], thereby further supporting our approach as a more viable option for IBD management. Although AA-induced colitis mimics the hallmarks of human IBD, it would be useful to corroborate the positive effects of ES and Au@ES in future investigations using, for example, ex vivo systems, as it is known that interspecies differences may make it difficult to predict treatment outcome of strategies validated in in vivo models [57]. This approach could also be useful in identifying the putative modulation of intestinal epithelial barrier function, which is central to IBD pathology.

The myenteric plexus neural network plays a vital role in the reflex regulation of contractile activities in the intestinal external musculature [6]. Intestinal ischemia/reperfusion in the digestive tract leads to a substantial loss of myenteric and submucosal plexus neurons, resulting in changes in neuron density and size, influencing intestinal motility [58]. In Crohn’s disease, transmural inflammation is associated with enteric nervous system lesions, such as hypertrophy and hyperplasia of neurons, an irregular increase in the number of nerve fibers, ganglia, and an augmented number of glial cells. The increase in the number of cells may reflect the severity of inflammation. Although the myenteric plexus is a crucial player in IBD pathology, few studies include its monitoring. Treatment with ES 50 mg/kg but more strikingly Au@ES 50 mg/kg strongly reduced the numbers of enteric glial cells, signifying that the benefits verified in the colon of mice also imply an impact on the myenteric plexus. To our knowledge, this is the first study indicating that ES and biosynthetic AuNPs may have this effect. Beyond its anti-inflammatory role, ES is known to be neuroprotective in other instances [10], which agrees with our results in terms of glial cell preservation.

## 5. Conclusions

We proved that ES and Au@ES applied in vivo diminished the inflammatory parameters of mice with AA-induced colitis. ES displayed a positive effect on the histopathology of the colon, clearly mediated by antioxidant and anti-inflammatory activities. The same effect was observed with Au@ES at lower doses, or more potently with equivalent doses of ES. For the first time, we show that both ES and Au@ES influence the myenteric plexus, boosting the beneficial effects on induced colitis, which may have a very interesting long-lasting effect. It is very apparent that the green synthesis of AuNPs in ES extract confers a synergistic, stronger impact, indicating that this is a very promising strategy for the treatment of IBD.

## Figures and Tables

**Table 1:** Experimental groups used in the study.

Groups	Description
Control	Administration of 0.9% saline solution in a volume of 0.8 mL rectally
Colitis group	Administration of 8% AA in a volume of 0.8 mL rectally
Dexamethasone group	Intraperitoneal (IP) pretreatment with dexamethasone 0.2 mL/30 g, 1 h before the induction of Crohn’s Disease (CD) with 8% AA solution, 0.8 mL rectally
ES group (25, 50 or 100 mg/kg)	Oral pretreatment with ES extract, 1 h before CD induction with 8% AA solution, 0.8 mL rectally
Au@ES group (25, 50 or 100 mg/kg)	Oral pretreatment with Au@ES, at a dose corresponding to 25 mg extract/kg, 1 h before disease induction with 8% AA, 0.8 mL rectally

**Table 2:** Inflammation score by macroscopic and microscopic analysis.

Microscopic Criteria	Score
Loss of mucosal architecture	0–3
Cell infiltration	0–3
Thickening of the muscle layer	0–3
Crypt abscess formation	0–1
Absence of goblet cells	0–1
Macroscopic criteria	Score
Normal appearance	0
Focal hyperemia, no ulcer	1
Ulceration without hyperemia or thickening of the colon wall	2
Ulceration with inflammation in one area	3
Ulceration with inflammation in two or more areas	4
Main lesion extending one centimeter along the colon	5
Main lesion extending 2 cm or more along the colon; one point is added for each additional centimeter	6–10

**Table 3:** Effect of ES treatment, at the dose of 25 (n = 6) and 50 mg/kg (n = 6) or dexamethasone (n = 6) on total macroscopic inflammation scores in the colon of mice with colitis induced by 8% AA (n = 6). Results are expressed as median (minimum–maximum). Data were analyzed using the Kruskal–Wallis test.

Experimental Group	Macroscopic Score
Control	0 (0–1)
8% AA	3 (1–4) ^a^
Dexamethasone	0.5 (0–2) ^b^
ES 25 mg/kg	1 (0–3) ^b^
ES 50 mg/kg	1 (0–3) ^b^

^a^ *p* < 0.05 vs. control group; ^b^ *p* < 0.05 vs. colitis group.

**Table 4:** Effect of Au@ES treatment, at the dose of 25 mg/kg (n =6), 50 mg/kg (n = 6), and 100 mg/kg (n = 6) or dexamethasone (n = 6) on total macroscopic inflammation scores in the colon of mice with colitis induced by 8% AA (n = 6). Results are expressed as median (minimum–maximum). Data were analyzed using the Kruskal–Wallis test.

Experimental Group	Macroscopic Score
Control	0 (0–1)
8% AA	3 (1–4) ^a^
Dexamethasone	0.5 (0–2) ^b^
Au@ES 25 mg/kg	2 (0–3) ^b^
Au@ES 50 mg/kg	1 (0–2) ^b^
Au@ES 100 mg/kg	2 (2–3)

^a^ *p* < 0.05 vs. control group; ^b^ *p* < 0.05 vs. colitis group.

**Table 5:** Effect of ES treatment at the dose of 25 mg/kg (n =6), 50 mg/kg (n = 6), and 100 mg/kg (n = 6) or dexamethasone (n = 6) on the microscopic scores of inflammations in the colon of mice with induced colitis induced by 8% AA (n = 6), according to the criteria of [8]. Results are expressed as median (minimum–maximum). Data were analyzed using the Kruskal–Wallis test.

Experimental Group	Microscopic Score
Control	0 (0–1)
8% AA	3 (1–3) ^a^
Dexamethasone	1 (0–2) ^b^
ES 25 mg/kg	2 (0–3)
ES 50 mg/kg	1 (0–2) ^b^
ES 100 mg/kg	2 (0–3)

^a^ *p* < 0.05 vs. control group; ^b^ *p* < 0.05 vs. colitis group.

**Table 6:** Effect of Au@ES treatment, at the dose of 25 mg/kg (n = 6), 50 mg/kg (n = 6), and 100 mg/kg (n = 6) or dexamethasone (n = 6) on the microscopic scores of inflammation in the colon of mice with AA-induced colitis, according to [8]. Results are expressed as median (minimum–maximum). Data were analyzed using the Kruskal–Wallis test. ^a^ *p* < 0.05 vs. control group; ^b^ *p* < 0.05 vs. colitis group.

Experimental Group	Microscopic Score
Control	0 (0–1)
8% AA	3 (1–3) ^a^
Dexamethasone	1 (0–2) ^b^
Au@ES 25 mg/kg	1 (0–3) ^b^
Au@ES 50 mg/kg	1 (0–2) ^b^
Au@ES 100 mg/kg	2 (0–3)

^a^ *p* < 0.05 vs. control group; ^b^ *p* < 0.05 vs. colitis group.

## Data Availability

Data will be made available upon reasonable request. The data are not publicly available as the institutional data repositories are being set up.

## Supplementary Materials

The following supporting information can be downloaded at https://www.mdpi.com/article/10.3390/antiox13080884/s1, Table S1: Effect of *E. selaginoides* (ES) extract and biosynthesized gold nanoparticles (Au@ES) on myeloperoxidase (MPO) levels in the colon of mice with experimental colitis induced with 8% acetic acid; Table S2: Effect of ES and Au@ES on reduced glutathione levels (GSH) in the colon of mice with experimental colitis; Table S3: Effect of ES and Au@ES on malondialdehyde (MDA) levels in the colon of mice with experimental colitis.
