# Supplementary material for: Exploring the Therapeutic Potential of Green-Synthesized Gold Nanoparticles and Ericaria selaginoides Extract for Inflammatory Bowel Disease
Source: Antioxidants (Basel). 2024 Jul 23;13(8):884. doi: 10.3390/antiox13080884 (PMC11351725; doi:10.3390/antiox13080884)
Supplement: Supplementary file 1 [file antioxidants-13-00884-s001.zip › antioxidants-3047222-supplementary.pdf]

SUPPLEMENTARY MATERIALS

Table S1. Effect of *E. selaginoides* (ES) extract and biosynthesized gold nanoparticles (Au@ES) on myeloperoxidase (MPO) levels in the colon of mice with experimental colitis induced with 8% acetic acid. Results are expressed in average ± standard error of the mean (SEM), in nmol/g tissue. The results were analysed with One-way ANOVA followed by Bonferroni test. [a] $p < 0.05$ versus control group; [b] $p < 0.05$ versus colitis group (acetic acid 8%).

EXPERIMENTAL GROUP	MPO nmol/g Tissue (Average ± SEM)
Control	10.65 ± 1.3
Acetic acid 8%	30.43 ± 2.9[a]
Dexamethasone	11.76 ± 2.1[b]
ES 25 mg/kg	21.97 ± 2.4
ES 50 mg/kg	9.77 ± 1.2[b]
ES 100 mg/kg	27. 63 ± 2.8
Au@ES 25 mg/kg	15.13 ± 1.9[b]
Au@ES 50 mg/kg	11.11 ± 1.7[b]
Au@ES 100 mg/kg	29.19 ± 2.9

Table S2. Effect of ES and Au@ES on reduced glutathione levels (GSH) in the colon of mice with experimental colitis. Results are expressed in average ± standard error of the mean (SEM), in mg of NPSH/g tissue. The results were analysed with One-way ANOVA followed by Bonferroni test. [a] $p < 0.05$ versus control group; [b] $p < 0.05$ versus colitis group (acetic acid 8%).

EXPERIMENTAL GROUP	NPSH nmol/g Tissue (Average ± SEM)	EXPERIMENTAL GROUP	NPSH/g Tissue (Average ± SEM)
Control	58.92 ± 6.9	Control	67.44 ± 5.5
Acetic acid 8%	28.24 ± 5.9[a]	Acetic acid 8%	28.24 ± 5.9[a]
Dexamethasone	55.86 ± 4.5[b]	Dexamethasone	55.86 ± 4.5[b]
ES 25 mg/kg	34.04 ± 4.4	Au@ES 25 mg/kg	50.29 ± 4.1[b]
ES 50 mg/kg	53.34 ± 2.1[b]	Au@ES 50 mg/kg	55.55 ± 3.8[b]
ES 100 mg/kg	31.22 ± 2.8	Au@ES 100 mg/kg	40.67 ± 6.1

Table S3. Effect of ES and Au@ES on malondialdehyde (MDA) levels in the colon of mice with experimental colitis. Results are expressed in average ± standard error of the mean (SEM), in nmol/g tissue. The results were analysed with One-way ANOVA followed by Bonferroni test. [a] $p < 0.05$ versus control group; [b] $p < 0.05$ versus colitis group (acetic acid 8%).

EXPERIMENTAL GROUP	MBA (nmol/g Tissue) (Average ± SEM)
Control	26.48 ± 3.0
Acetic acid 8%	65.85 ± 6.8[a]
Dexamethasone	27.41 ± 4.6[b]
ES 25 mg/kg	45.26 ± 4.8
ES 50 mg/kg	31.30 ± 3.6[b]
ES 100 mg/kg	52.36 ± 6.0
Au@ES 25 mg/kg	34.09 ± 1.0[b]
Au@ES 50 mg/kg	27.25 ± 4.4[b]
Au@ES 100 mg/kg	49.67 ± 5.9
